# Factors affecting log-transformed muscle ^137^Cs concentrations in wild boars in Fukushima Prefecture over 14 years

**DOI:** 10.1371/journal.pone.0344189

**Published:** 2026-03-25

**Authors:** Hisashi Komatsu, Shiori Ikushima

**Affiliations:** 1 Fukushima Prefectural Centre for Environmental Creation, Miharu, Fukushima, Japan; 2 Fukushima Regional Collaborative Research Center, National Institute for Environmental Studies, Miharu, Fukushima, Japan; University of South Carolina, UNITED STATES OF AMERICA

## Abstract

The March 2011 Fukushima Daiichi Nuclear Power Plant accident resulted in extensive radiocesium contamination of forest ecosystems. Wild boars (*Sus scrofa*) are a key indicator species because of their high radiocesium accumulation; however, long-term spatiotemporal patterns and biological drivers of contamination have not been fully evaluated using a prefecture-wide dataset. We analyzed monitoring data from 3,609 wild boars collected across Fukushima Prefecture over 14 years (FY2011–FY2025). By integrating individual-level measurements with spatial soil deposition data, we fitted three mixed-effects models to ln-transformed muscle ^137^Cs concentrations to quantify regional ecological half-lives, assess dietary influence using stomach-content ^137^Cs where available, and evaluate associations with biological attributes such as sex and growth stage. Ecological half-lives of muscle ^137^Cs ranged from 3.0 to 9.2 years across regions, shorter than the physical half-life of 30.1 years, with the most rapid decline observed in Hamadori. Although long-term decreases were evident, a transient increase occurred in Nakadori in FY2022, indicating the influence of localized ecological variability. In individuals with paired stomach-content measurements, muscle ^137^Cs increased with stomach-content ^137^Cs, supporting a dietary pathway for short-term variation; however, paired stomach-content data were limited in some regions. Growth stage was significantly associated with muscle ^137^Cs, with evidence consistent with higher adult burdens relative to younger animals. These results show that radiocesium dynamics in wild boars reflect regional recovery processes, dietary pathways, and biological attributes. Our findings emphasize the value of long-term, multi-variable monitoring frameworks for assessing radiological risk and ecosystem recovery in wildlife inhabiting post-accident landscapes.

## Introduction

The March 2011 accident at the Fukushima Daiichi Nuclear Power Plant (FDNPP) released substantial quantities of radiocesium (^134^Cs and ^137^Cs) into the environment [1,2]. Although extensive decontamination has reduced radiation levels in residential and agricultural areas, forest ecosystems—which cover approximately 70% of Fukushima Prefecture—remain a major long-term reservoir of radiocesium [3,4]. Within these forests, radiocesium continues to cycle among soil, vegetation, and wildlife, complicating assessments of environmental recovery and exposure risk [5,6].

Wild boars (Sus scrofa) are a particularly informative indicator species because their omnivorous diet and frequent rooting in forest soils can lead to high radiocesium accumulation, sometimes exceeding Japan’s regulatory limit for human consumption (100 Bq/kg) [7,8]. Monitoring has consistently shown strong regional contrasts in muscle radiocesium that reflect initial deposition patterns [9,10]. However, temporal trends in wild boars do not follow physical decay alone and can exhibit substantial variability, including localized increases reported even a decade after the accident [11].

Despite the availability of long-term monitoring records, the drivers of these spatiotemporal patterns remain incompletely resolved. Many previous studies have been descriptive, geographically limited, or based on smaller datasets, making it difficult to quantify the relative contributions of time since accident, regional contamination, dietary intake, and biological attributes such as sex and age or body size [12–14]. Moreover, evidence regarding age-dependent accumulation is mixed: some studies suggest higher concentrations in juveniles due to high intake rates relative to body mass, whereas others suggest higher burdens in adults consistent with cumulative exposure or age-related differences in habitat use and foraging [12]. In addition, ecological half-lives of radiocesium in wild boars have rarely been quantified using a prefecture-wide dataset spanning more than a decade with model-based inference.

Here, we analyzed monitoring data from a total of 3,941 wild boars collected across Fukushima Prefecture over 14 fiscal years (FY2011-FY2025), with 3,609 individuals included in the final statistical analysis after data screening. By linking individual-level measurements to spatial soil deposition data and applying linear mixed-effects models, we aimed to (1) quantify region-specific ecological half-lives of muscle ^137^Cs, (2) evaluate dietary influence using paired stomach-content ^137^Cs where available, and (3) assess demographic patterns, including growth-stage differences in both contamination levels and temporal decline rates. This integrative approach provides a model-based framework for identifying key factors shaping radiocesium dynamics in wildlife inhabiting post-accident forest landscapes.

## Materials and methods

### Sampling and data collection

We monitored a total of 3,941 wild boars collected across Fukushima Prefecture from April 2011 to August 2025 (FY2011-FY2025). Of these, 3,609 individuals collected through July 2025 were included in the final statistical analysis after excluding those with missing data (S1 Dataset). All animals were culled under official pest control or specified wildlife management programs administered by Fukushima Prefecture [7]. Licensed hunters performed euthanasia using firearms in accordance with the Wildlife Protection and Hunting Management Law of Japan. Muscle samples (approximately 500 g) were collected mainly from the quadriceps femoris.

Radiocesium (^134^Cs and ^137^Cs) concentrations were measured at the Radiation Survey Division of the Fukushima Prefectural Government using high-purity germanium detectors (GC3018, GC4020, and GR4521; Canberra Japan, Tokyo, Japan). The counting time was set to 3,600 s, and the detection limit for ^137^Cs was 4–9 Bq/kg. All activity concentrations are reported as Bq/kg wet weight and were decay-corrected to the animal capture date using the physical half-life of ^137^Cs (30.1 years).

Wild boar samples used in this study were obtained from animals legally captured within Fukushima Prefecture, Japan. Captures were conducted under the Specified Wildlife Management Capture Program and the Nuisance Wildlife Control Program, administered by Fukushima Prefecture and its municipalities, and carried out by licensed hunters belonging to the prefectural hunters’ association in accordance with the Wildlife Protection and Hunting Management Law of Japan and relevant local regulations.

Euthanasia of captured animals was performed exclusively by licensed hunters as part of these official management programs and not for research purposes. Researchers did not participate in the capture or euthanasia of animals. Only post-mortem tissue samples were collected opportunistically after euthanasia had been completed.

Sample handling followed the guidelines of the Mammal Society of Japan. Because the study relied solely on post-mortem materials obtained independently of the research and required no experimental manipulation, anesthesia, or euthanasia by the investigators, formal approval from an institutional animal ethics committee was not required. The Fukushima Prefectural Centre for Environmental Creation does not operate an ethics review system for studies of this type; therefore, no institutional approval number was issued.

#### No endangered or protected species were used in this study.

### Spatial analysis and soil contamination

**Fig 1:**
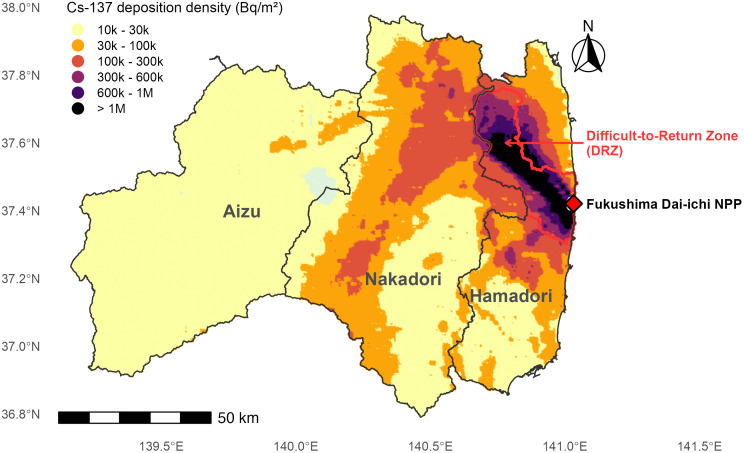
Map of the study area in Fukushima prefecture and soil ^137^Cs deposition.

To evaluate the influence of initial environmental contamination, we linked each wild boar capture location to a spatial estimate of soil radiocesium deposition. Using the scipy.spatial.cKDTree function in Python [15], we performed nearest-neighbor matching to assign a ^137^Cs deposition density value (kBq/m^2^; reference date June 2012) from the Japan Atomic Energy Agency (JAEA) deposition dataset to each capture site [16]. For visualization in Fig 1, the deposition heat map was generated separately using publicly available airborne monitoring survey outputs distributed via the NRA monitoring portal under the Government of Japan Standard Terms of Use (compatible with CC BY 4.0). For subsequent analyses, the study area was divided into four regions based on administrative boundaries and contamination status: the Difficult-to-Return Zone (DRZ), Hamadori (excluding the DRZ), Nakadori, and Aizu (Fig 1).

The study area was categorized into four regions based on administrative boundaries and contamination status: Hamadori (excluding the DRZ), Nakadori, Aizu, and the Difficult-to-Return Zone (DRZ). The background heat map shows soil ^137^Cs deposition density (kBq/m^2^) as of June 2012, based on airborne monitoring survey outputs distributed via the NRA monitoring portal (https://radioactivity.nra.go.jp/en) under the Government of Japan Standard Terms of Use (compatible with the CC BY 4.0 license). The base map was created by the authors using the National Land Numerical Information (Administrative Zones) provided by the Ministry of Land, Infrastructure, Transport and Tourism of Japan (https://nlftp.mlit.go.jp/ksj/index.html), which is freely available for use under the CC BY 4.0 license.

### Statistical analysis

We used linear mixed-effects models to identify factors associated with radiocesium concentrations in wild boar muscle. All analyses were performed in R (v4.3.1) [17] using the lme4 and lmerTest packages [18,19] (S2 Code). The response variable was the natural logarithm of muscle ^137^Cs concentration (logMus, ln [Bq/kg wet]). To account for spatial clustering, municipality was included as a random intercept.

We fitted three models to address distinct ecological questions. Model 1 (spatiotemporal trend) assessed region-specific temporal change by including fiscal year (continuous; Year_num = FY − 2011), region, the region × year interaction, and log-transformed soil ^137^Cs deposition (logSoil). Model 2 (dietary influence) evaluated the association between muscle and stomach-content ^137^Cs in the subset with paired measurements, using log-transformed stomach-content ^137^Cs, year, region, and logSoil as fixed effects. Model 3 (demographic attributes) assessed associations with sex and growth stage (juvenile, subadult, adult) while adjusting for year, region, and logSoil.

To test whether temporal loss rates differed by sex or growth stage, we fitted extensions of Model 3 that included Year_num × Sex or Year_num × Growth stage terms, while retaining the region × year interaction and controlling for logSoil, with municipality as a random intercept.

Statistical significance of fixed effects was evaluated using Type III ANOVA with Satterthwaite’s approximation for degrees of freedom (Wald F-tests). For Model 3, growth-stage differences were further examined using estimated marginal means (emmeans) and Tukey-adjusted pairwise comparisons. Ecological half-lives (*T*_eco_) were calculated from the estimated annual slope (β) as *T*_eco_ = ln(2)/|β|; confidence intervals for *T*_eco_ were obtained by transforming the corresponding confidence limits of the slope estimates [20].

## Results

### Spatiotemporal trends and ecological half-lives

**Fig 2:**
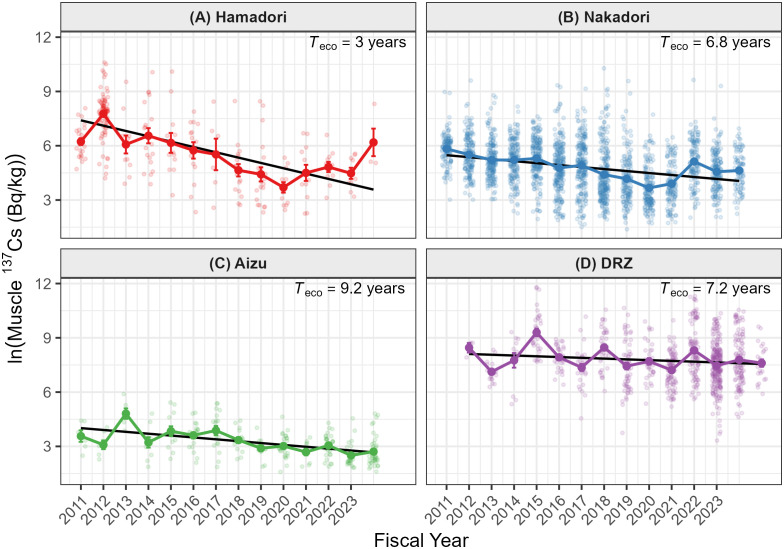
Spatiotemporal trends of radiocesium concentrations in wild boar muscle (FY2011–FY2023). Boxplots show annual ^137^Cs concentrations (Bq/kg wet weight) for **(A)** Hamadori, **(B)** Nakadori, and **(C)** Aizu. Boxes represent the interquartile range (IQR), horizontal lines indicate medians, and black dots indicate means. The y-axis is displayed on a log10 scale. Values of *T*_eco_ shown in the upper-right of each panel are ecological half-lives estimated from the mixed-effects model (Model 1). An increase in Nakadori in FY2022 is visible.

**Fig 3:**
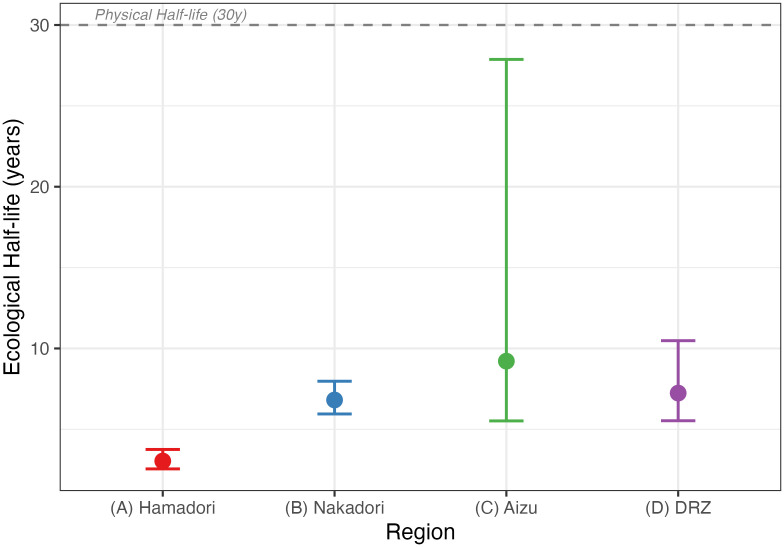
Comparison of ecological and physical half-lives of ^137^Cs. Ecological half-lives (*T*_eco_; bars) estimated for each region from the mixed-effects model (Model 1) are shown together with the physical half-life of ^137^Cs (30.1 years; dashed horizontal line). Error bars indicate 95% confidence intervals.

A linear mixed-effects model detected significant temporal declines in muscle ^137^Cs concentrations across all regions (Fig 2; Table 1, Model 1). The Fiscal Year × Region interaction was significant (p < 0.001), indicating that the rate of decline differed among regions. Region-specific ecological half-lives (*T*_eco_) estimated from the model ranged from 3.0 to 9.2 years and were shorter than the physical half-life of ^137^Cs (30.1 years) (Fig 3). The shortest *T*_eco_ was observed in Hamadori (3.0 years), followed by Nakadori (6.8 years) and Aizu (9.2 years). In Nakadori, muscle ^137^Cs concentrations increased in FY2022 relative to adjacent years (Fig 2B).

**Table 1:** Region-specific decline rates (Slope) and ecological half-lives (*T*_eco_) of muscle ^137^Cs in wild boars (Model 1).

Region	Slope	*T*_eco_ (years)	95% CI (lower)	95% CI (upper)
Aizu	−0.08	9.2	5.5	27.9
Nakadori	−0.1	6.8	5.9	8.0
Hamadori	−0.23	3	2.6	3.8
DRZ	−0.1	7.2	5.5	10.5

*T*_eco_ was calculated as ln(2)/|Slope|. Slopes are the estimated annual effects of fiscal year (Year_num = FY − 2011) on ln-transformed muscle ^137^Cs from Model 1. The 95% CI for *T*_eco_ was derived by transforming the corresponding CI of the slope estimate.

### Dietary influence

**Fig 4:**
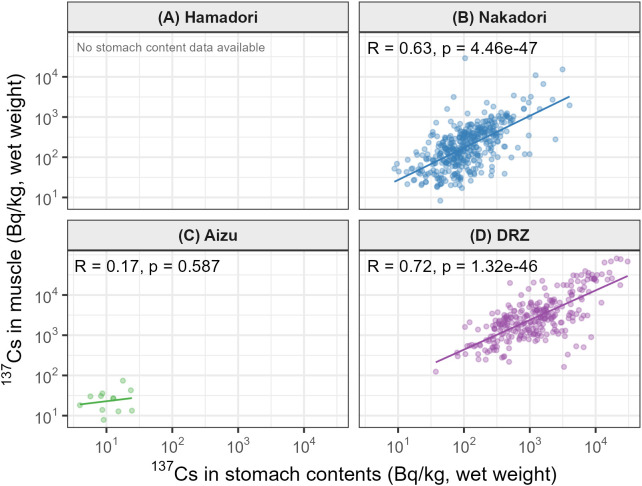
Relationship between radiocesium in stomach contents and muscle tissue. Scatter plots show the association between ln-transformed ^137^Cs concentrations in stomach contents (x-axis) and ln-transformed ^137^Cs concentrations in muscle tissue (y-axis) for each region: **(A)** Hamadori, **(B)** Nakadori, **(C)** Aizu, and (D) the Difficult-to-Return Zone (DRZ). Solid lines indicate ordinary least squares fits. No individuals from Hamadori had paired stomach-content measurements; therefore, the Hamadori panel is shown without data points. In Aizu, the number of paired observations was limited (N = 10).

Muscle ^137^Cs was positively associated with stomach-content ^137^Cs in the paired subset (Fig 4). Region-specific slope estimates from the interaction model indicated significant positive associations in Nakadori and the DRZ (both p < 0.001), whereas the estimate for Aizu was imprecise and not significant (p = 0.95; N = 10) (Fig 4). The Region × stomach ^137^Cs interaction was not significant (p = 0.29), providing no evidence that the strength of the stomach–muscle association differed among regions. Paired stomach-content measurements were not available for Hamadori because stomach sampling was not included in the monitoring protocol for that region (Fig 4).

### Growth stage and sex effects

**Fig 5:**
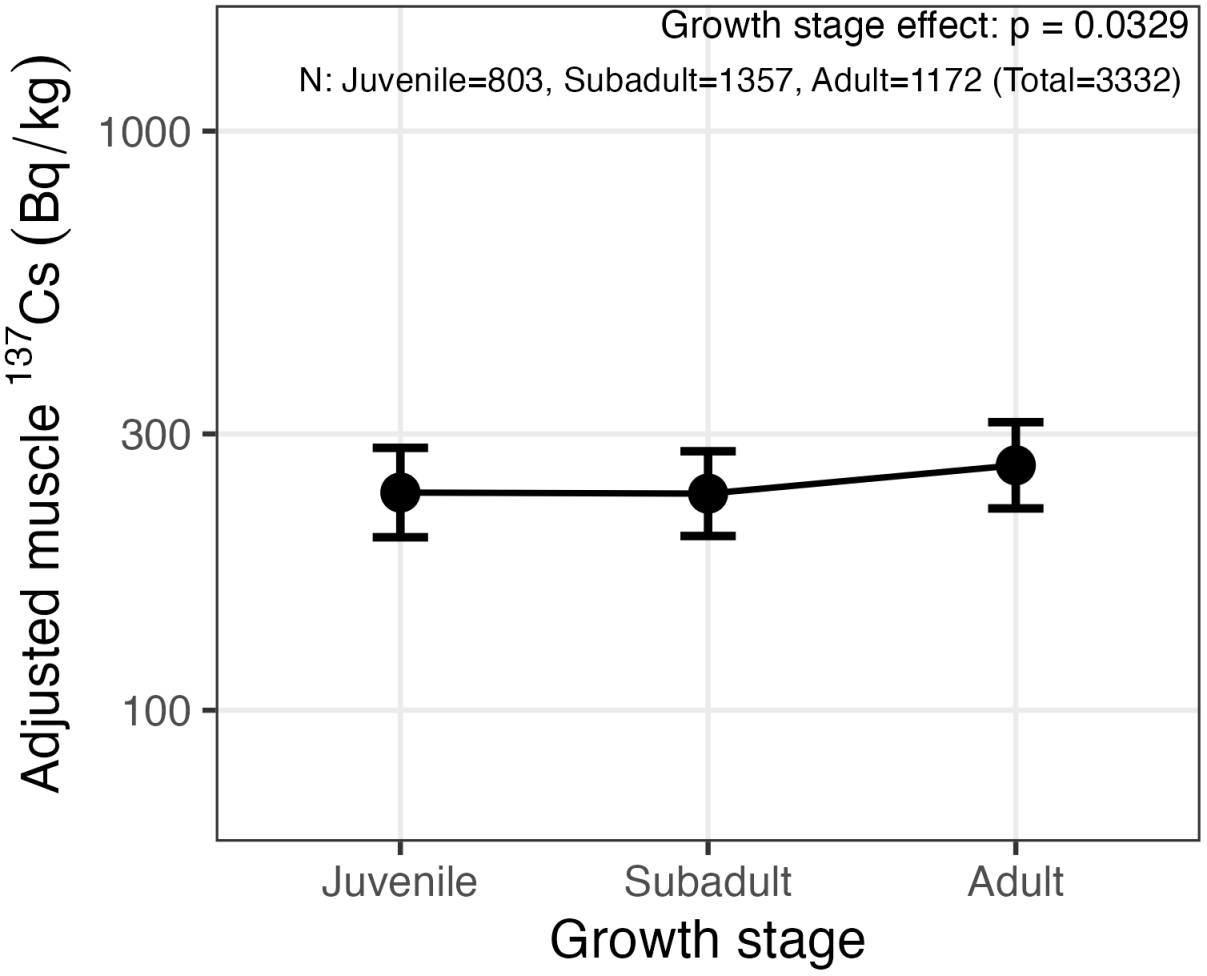
Growth-stage differences in muscle ^137^Cs concentrations. Points show estimated marginal means of muscle ^137^Cs for juvenile, subadult, and adult wild boars from the mixed-effects model adjusting for fiscal year, region, soil deposition, and sex with municipality as a random intercept. Values are back-transformed to Bq/kg (wet weight), with 95% confidence intervals. The y-axis is shown on a log10 scale. Growth stage showed a significant overall effect (Type III test, p = 0.03). Sample sizes are shown in the panel.

Sex was not associated with muscle ^137^Cs concentrations after adjustment for fiscal year, region, soil deposition, and municipality-level clustering (Model 3; Type III test, p = 0.90). Growth stage showed a significant overall association with muscle ^137^Cs in this model (Type III test, p = 0.03; Fig 5). Tukey-adjusted pairwise comparisons indicated that the adult–subadult contrast was significant (p = 0.04), whereas the adult–juvenile contrast was not significant after adjustment (p = 0.12).

To evaluate whether temporal decline rates differed among demographic groups, we fitted extensions of Model 3 that included interaction terms between fiscal year (Year_num) and sex or growth stage. The Year_num × Sex interaction was not significant (Type III test, p = 0.515), providing no evidence of sex-specific decline rates. In contrast, the Year_num × growth stage interaction was significant (Type III test, p < 0.001), indicating stage-specific temporal slopes. Estimated annual slopes (ln scale) were −0.191 (juvenile), −0.241 (subadult), and −0.220 (adult), corresponding to ecological half-lives of 3.6, 2.9, and 3.1 years, respectively (Table 2).

**Table 2:** Stage-specific temporal slopes and ecological half-lives (*T*_eco_) of muscle ^137^Cs.

Growth stage	N	Slope	95% CI (Slope)	*T*_eco_ (years)	95% CI (*T*_eco_)
Juvenile	803	−0.191	−0.239 to −0.143	3.6	2.9 to 4.9
Subadult	1357	−0.241	−0.286 to −0.196	2.9	2.4 to 3.5
Adult	1172	−0.22	−0.265 to −0.175	3.1	2.6 to 4.0

Slope represents the annual effect of fiscal year (Year_num = FY − 2011) on ln-transformed muscle ^137^Cs for each growth stage, estimated from a linear mixed-effects interaction model including Year_num × growth stage and Region × Year_num terms, with log-transformed soil ^137^Cs deposition (logSoil) and sex as covariates and municipality as a random intercept. Ecological half-lives were calculated as *T*_eco_ = ln(2)/|Slope|. Confidence intervals for *T*_eco_ were obtained by transforming the corresponding confidence limits of the slope estimates.

## Discussion

### Ecological half-lives and regional recovery

Muscle ^137^Cs concentrations in wild boars declined across Fukushima over the 14-year monitoring period, and region-specific ecological half-lives (*T*_eco_) were estimated to be 3.0–9.2 years (Fig 3; Table 1). Decline rates differed significantly among regions (Year × Region interaction), with the shortest *T*_eco_ in Hamadori (3.0 years) and longer values in Nakadori (6.8 years) and Aizu (9.2 years). All estimated ecological half-lives were shorter than the physical half-life of ^137^Cs (30.1 years), indicating that ecological processes affecting bioavailability contributed to the observed declines in addition to radioactive decay. Notably, regional differences in *T*_eco_ persisted even after adjusting for soil ^137^Cs deposition, suggesting that unmeasured region-specific factors (e.g., soil properties and hydrology, decontamination intensity, and habitat/diet composition) likely influence the long-term bioavailability of radiocesium.

The relatively short *T*_eco_ values may reflect processes that reduce bioavailable radiocesium, including strong fixation of Cs in micaceous soils [21] and redistribution or removal of surface contamination under precipitation-driven erosion and runoff [6]. In the Difficult-to-Return Zone (DRZ), absolute concentrations remained higher than in other regions, but the estimated *T*_eco_ (7.2 years) still indicated an overall decline, consistent with gradual reductions in availability even in highly contaminated landscapes.

### Age- or growth-stage patterns

Growth stage appeared to modify the temporal dynamics of muscle ^137^Cs, as indicated by stage-specific annual slopes and corresponding ecological half-lives estimated from the interaction model (Table 2). These differences likely reflect a combination of exposure history and age-dependent ecology rather than physiological clearance alone. Adults may more frequently exploit soil-associated resources (e.g., roots and tubers) that can retain radiocesium longer, whereas subadults may experience stronger growth-related dilution and dietary transitions during maturation, producing a faster apparent decline. Overall, the stage dependence observed here suggests that incorporating growth stage can improve interpretation of long-term monitoring trends and reduce confounding when comparing temporal trajectories across populations or regions (Table 2).

### The FY2022 increase in Nakadori and dietary pathways

The FY2022 increase in Nakadori suggests that wild boar ^137^Cs burdens can be shaped by short-term ecological variability even long after the initial deposition. In the absence of new atmospheric inputs, plausible drivers include shifts in resource use (e.g., increased reliance on radiocesium-retentive foods such as fungi or other forest products during particular years) [22] and changes in the composition of the sampled population through movement from more contaminated areas, including the nearby Difficult-to-Return Zone [11]. The positive association between stomach-content and muscle ^137^Cs supports diet as a proximate pathway for short-term variation [23], but inference is constrained by uneven availability of paired stomach data (unavailable in Hamadori and sparse in Aizu), which limits regional comparison. Discriminating between dietary versus movement-based explanations will require complementary information such as seasonal diet indicators (e.g., mast/fungi proxies) and movement data. More generally, this event cautions against interpreting long-term monitoring as a strictly monotonic decline and highlights the value of integrating biological and ecological context into trend assessment.

### Implications for monitoring and management

Our results highlight the limitations of relying on soil deposition alone to predict individual muscle ^137^Cs burdens, particularly in lower-contamination settings where biological variability can dominate. Limited paired stomach-content data in some regions (unavailable in Hamadori and sparse in Aizu) further indicate that dietary pathways may be difficult to quantify uniformly across the prefecture.

The FY2022 increase in Nakadori underscores that temporal patterns are not necessarily monotonic and that ecological variability can generate transient increases. Monitoring programs may therefore benefit from sustained long-term sampling and from integrating environmental gradients with biological attributes—potentially including growth stage–specific decline rates—and dietary indicators within mixed-effects modeling frameworks. In this context, ongoing diet analyses will be valuable for strengthening mechanistic interpretation; expanding dietary information (e.g., stomach contents and/or complementary dietary indicators) alongside radiocesium measurements should allow more direct evaluation of how resource use and seasonal shifts contribute to short-term variability in muscle ^137^Cs.

## Conclusion

Longitudinal analysis of 3,609 wild boars over 14 years showed that muscle ^137^Cs concentrations declined across Fukushima Prefecture, with region-specific ecological half-lives (*T*_eco_ = 3.0–9.2 years) shorter than the physical half-life of ^137^Cs. In the subset of individuals with paired measurements, muscle ^137^Cs was positively associated with stomach-content ^137^Cs, supporting a dietary contribution to short-term variation in individual burdens; however, paired stomach-content data were unavailable in Hamadori and sparse in Aizu, limiting regional inference. Mixed-effects models also indicated that growth stage was related to muscle ^137^Cs, and interaction analyses suggested that temporal decline rates differed among growth stages.

Temporal declines were not uniform across the prefecture. The increase observed in Nakadori in FY2022 illustrates that ecological variability can produce short-term departures from longer-term declining patterns even more than a decade after the accident. Together, these findings support sustained monitoring approaches that integrate environmental conditions with biological attributes (including growth stage–specific temporal trends) and dietary indicators within mixed-effects frameworks, to improve understanding of radiocesium dynamics and to inform wildlife management in post-accident landscapes.

## Supporting information

S1 Dataset Raw data of wild boar radiocesium concentrations. This file includes capture dates, locations, biological attributes, and ^137^Cs concentrations for all 3,941 individuals collected across Fukushima Prefecture between FY2011 and FY2025. A subset of 3,609 individuals with complete records was used for the final statistical analysis.
(CSV)

S2 Code R script for linear mixed models. The R code used to perform the statistical analyses and generate the results presented in this study (R).
(ZIP)
