# Supplementary material for: Factors affecting log-transformed muscle 137Cs concentrations in wild boars in Fukushima Prefecture over 14 years
Source: PLoS One. 2026 Mar 25;21(3):e0344189. doi: 10.1371/journal.pone.0344189 (PMC13016332; doi:10.1371/journal.pone.0344189)
Supplement: Attachment — Submitted filename: Response to Reviewers.docx [file pone.0344189.s003.docx]

Response to Reviewers

Manuscript ID: PONE-D-25-17137 (previous submission)
Title: Factors affecting log-transformed muscle ¹³⁷Cs concentrations in wild boars in Fukushima Prefecture over 12 years

Dear Academic Editor and Reviewers,

We sincerely thank the reviewers for their constructive and insightful comments, which highlighted that the initial submission was largely descriptive and required stronger statistical support. In response, we revised the study as follows:

1. re-analyzed the entire dataset (N = 3,941) using linear mixed-effects models to evaluate the effects of time, location, diet, and biological attributes;
2. estimated ecological half-lives for each region to quantify temporal decline rates;
3. evaluated growth-stage differences, showing a significant overall effect and evidence consistent with higher adult burdens; and
4. linked individual capture locations with spatial soil contamination data using a nearest-neighbor interpolation approach.

These changes strengthen the manuscript by adding explicit model-based estimates and hypothesis-driven tests. Below, we provide point-by-point responses. Reviewer comments are reproduced in italics, followed by our responses.

**Comment 1**

**(1)** The author may have conducted proper statistics, but there is insufficient reporting for the reader to evaluate the stats. The Cs activity concentration data are most likely log-normally distributed. The author has not addressed this and has not attempted to convince the reader that his use of ANOVA statistical tests are appropriate for data that are not normally distributed. If the data are log-normal then geometric means and geometric standard deviations might be appropriate presentations.

**Response:** We agree with the reviewer that the original statistical reporting was insufficient and that ¹³⁷Cs activity concentrations are approximately log-normally distributed. In the revised manuscript, we replaced the simple ANOVA with linear mixed-effects models and used a natural-log transformation of muscle ¹³⁷Cs (logMus, ln[Bq/kg]) to address the distributional properties of the data. We also expanded the statistical reporting (model structure, fixed effects, random effects, and inference approach) to enable evaluation of the analysis.

**Where to look:** Methods, Section 2.3 (Statistical Analysis); Results, Section 3.1–3.3 (model-based inference); Table 1 (Models 1–3).

**Comment 2**

**(2)** I did not see a data availability statement or a link to Supportive Information Files.

**Response:** We apologize for the oversight. We added a Data Availability Statement and prepared Supporting Information (S1 Dataset) containing the data used for the mixed-effects analyses.

**Where to look:** Data Availability Statement (manuscript front matter or end section, per journal format); Supporting Information: S1 Dataset.

**Comment 3**

**(3)** A figure is needed to show the location of the three study areas relative to the nuclear reactor. That figure would be even more informative if it included an overlay of ambient dose rates routinely supplied by the Japanese Government.

**Response:** We added a new map figure (Fig 1) showing the study regions (Hamadori, Nakadori, Aizu) and the Difficult-to-Return Zone (DRZ) relative to the FDNPP. The figure includes a spatial overlay of contamination (soil ¹³⁷Cs deposition) to visualize the contamination gradient.

**Where to look:** Fig 1 (map) and its legend; Methods, Section 2.2 (Study areas / spatial data).

**Comment 4**

**(4)** Citing of literature is inconsistent. Most citations are numerical, but some citations are by authors and year (i.e. lines 45, 57, 231, 256, 306 and 310). Some of the latter may not be in the Literature cited.

**Response:** We checked all in-text citations and the reference list and reformatted them consistently to the journal style. All citations in the text now correspond to entries in the reference list.

**Where to look:** Introduction + Discussion (formerly mixed-format citations); References section (full consistency check).

**Comment 5**

**(5)** The author documents a decrease in Cs activity concentrations over time, but does not calculate the rate of decrease. Is the decrease the same as the half-life of ¹³⁷Cs? Are the decreases the same in all three areas? Is the decrease the same as that documented for ambient dose rates provided by the Japanese government? Is there any evidence that ¹³⁷Cs is being retained in Fukushima wild boar longer than expected, perhaps similarly to the abundant published data for European boar contaminated from the Chernobyl accident?

**Response:** We agree this was an important point. We now quantify decline rates using a mixed-effects model (Model 1) that estimates region-specific annual slopes (on the ln scale) and converts them to ecological half-lives (Teco = ln(2)/|β|). Teco ranged from 3.0 to 9.2 years across regions and was shorter than the physical half-life of ¹³⁷Cs (30.1 years), indicating that observed decreases are not governed by physical decay alone and differ by region.

**Where to look:** Results, Section 3.1; Table 1 (region-specific slope and Teco); Fig 3 (Teco vs physical half-life); Discussion, Section 4.1.

**Comment 6**

**(6)** The author has a relatively large data set. Could you test to see if Cs loss rates differed among genders or age classes?

**Response:** Yes. We tested whether temporal loss rates differed by sex or growth stage by adding interaction terms with fiscal year (Year_num) to the mixed-effects framework, while retaining region-specific temporal trends (Region × Year_num), controlling for soil deposition (logSoil), and accounting for municipality-level clustering (random intercept). The Year_num × Sex interaction was not significant (Type III test, p = 0.52), indicating no evidence that temporal decline rates differed between males and females. In contrast, the Year_num × Growth stage interaction was significant (Type III test, p < 0.001), demonstrating that decline rates differed among growth stages. In addition, growth stage differed in adjusted mean levels in Model 3 (Type III test, p = 0.03; Fig 5), with Tukey-adjusted comparisons showing a significant adult–subadult difference (p = 0.04), while the adult–juvenile comparison was not significant after adjustment (p = 0.12). A single planned adult–juvenile contrast was marginally significant (p = 0.0497). These results are described in the revised Results and Discussion, and we provide stage-specific slopes and ecological half-lives in Table 2.

**Where to look:** Results, Section 3.3 (sex/stage tests + interaction results); Discussion, Section 4.2; Table 2 (stage-specific slopes and Teco); Fig 5 (adjusted means by growth stage).

**Comment 7**

(7) The Discussion section highlights several plausible reasons for the differences observed among the three areas. Those plausible reasons have been stated many times in prior publications. Unfortunately, your data does not advance the science by excluding some of those plausible reasons as the reason for your observations. You do not give the reader any data on other differences among the three study sites that might explain Cs behavior. Are differences in soil types, topography, precipitation, vegetation types, etc.?

**Response:**
We agree with the reviewer that site-specific environmental factors (e.g., soil type, topography, precipitation, vegetation) are plausible drivers of regional differences. However, these variables were not available at the individual capture-site level in our dataset; therefore, we cannot test them directly and now state this limitation explicitly in the revised Discussion (Sections 4.1–4.4).

Within the scope of the available data, we strengthened the Discussion by focusing on model-based inferences that help constrain interpretations. First, we incorporated spatial soil ^137^Cs deposition as a covariate (logSoil) in all models, allowing us to evaluate regional temporal trends after accounting for differences in initial contamination. The region-specific decline rates remained heterogeneous (significant Region × Year_num in Model 1; *p*<0.001), indicating that regional differences in temporal dynamics are not explained by deposition alone.

Second, we quantified the diet–muscle association using paired stomach-content measurements and documented the resulting data constraints (Hamadori: stomach sampling not conducted under the monitoring protocol; Aizu: very small paired sample size, *n*=10). We also tested for regional heterogeneity in the stomach–muscle relationship by fitting a Region × stomach ^137^Cs interaction; this interaction was not supported (*p*=0.29), providing no evidence that the stomach–muscle slope differs among regions within the paired subset.

**Where to look:** Discussion, Sections 4.1–4.4 (reduced speculation; explicit limitations; soil-adjusted regional trends; dietary subset constraints); Results, Sections 3.1–3.2.

**Comment 8**

**(8)** My major comment is that you have not used this data set to further our understanding of Cs dynamics in wild boar. What does your paper do to advance the science beyond what others have done using similar analyses of Cs concentrations over time in Fukushima boar?

**Response:** We believe the revised manuscript advances understanding by (i) quantifying region-specific ecological half-lives using a large prefecture-wide dataset over 12 years, (ii) identifying that growth stage modifies not only adjusted mean levels but also the estimated loss rates (stage-specific slopes and half-lives), and (iii) detecting a localized temporal anomaly (FY2022 increase in Nakadori) that cautions against assuming monotonic decline.

**Where to look:** Results, Sections 3.1 and 3.3; Table 1 (regional slopes/Teco) and Table 2 (stage-specific slopes/Teco); Discussion, Sections 4.1–4.3.

**Response to Reviewer #2**

**Comment 1**

The paper highlights several important unknowns regarding the bioaccumulation of radiocesium in animals, with a particular focus on wild boar, where observed patterns appear to differ between Europe and Japan. However, the manuscript reads more like a review than an original scientific article. As these ‘unknowns’ are already known. Additionally, while I have no objection to the use of tools such as ChatGPT for translation or language correction, the text reads as though it was entirely generated by such software (from the title to the subheadings) which detracts from the scientific tone and clarity.

**Response:** We revised the manuscript to emphasize original analyses directly derived from the monitoring dataset and reorganized the text into an original-article structure, focusing on model-based results rather than review-like narrative. We also revised phrasing and structure throughout to improve scientific tone and clarity.

**Where to look:** Overall structure (IMRaD); Results, Sections 3.1–3.3 (new analyses); revised Abstract and section headings (tone/clarity).

**Comment 2**

In terms of data, the only results presented are annual mean concentrations of 134Cs+137Cs in wild boar. It is unclear how this dataset “demonstrates that the concentrations of radioactive cesium in wild boar muscle tissues in Fukushima Prefecture since the FDNPP accident are the result of a complex interplay among initial soil deposition, environmental dynamics, ecological factors, and human activities” …

**Response:** We moved beyond annual means by analyzing individual-level data using mixed-effects models that quantify associations with time, region-specific temporal trends, soil deposition, diet (paired stomach content), and demographic attributes. We revised the wording of conclusions to reflect what is directly supported by these analyses.

**Where to look:** Results, Sections 3.1–3.3; Methods, Section 2.3 (models); revised concluding statements (Abstract/Discussion/Conclusion).

**Comment 3**

There is no detailed statistical or spatial analysis to support this conclusion. Presenting annual averages in box plots for three broad regions over 13 years does little to advance our understanding…

**Response:** We added detailed statistical analyses using mixed-effects models with explicit model structures, covariates, and inference. We also strengthened the spatial context by adding a map figure and by linking capture locations to soil deposition data used as covariates.

**Where to look:** Methods, Section 2.3 (mixed-effects models, inference); Fig 1 (map); Results, Sections 3.1–3.3.

**Comment 4**

Another issue is that the authors assume that the reader will understand all of these geological locations. A detailed map is needed with wild boar samples plotted is needed. Deposition map of Fukushima will also help readers.

**Response:** We added a map (Fig 1) showing the study regions and contamination gradient (soil deposition overlay) to provide geographic context for readers unfamiliar with the area.

**Where to look:** Fig 1 and legend; Methods, Section 2.2 (study area definitions).

**Comment 5**

Paper needs to be reorganized and new scientific findings new to be considered… (citations suggested)

**Response:** We reorganized the manuscript into a standard IMRaD format and reviewed the suggested studies in revising the Introduction and Discussion. We clarify our study’s contribution as a prefecture-wide long-term analysis through FY2023 with additional tests on growth-stage–specific decline rates and the FY2022 anomaly in Nakadori.

**Where to look:** Introduction (positioning vs prior work); Discussion, Sections 4.1–4.3; Results, Sections 3.1 and 3.3.

**Minor issues**

-M&M has things that, I would not consider M&M (first 2 paragraphs)
-number of samples should be stated in abstract and M&M

**Response:** We streamlined the Materials and Methods section to include only methodological content and stated the sample size (N = 3,941) in both the Abstract and Methods.

**Where to look:** Abstract (sample size); Methods, Section 2.1–2.3 (streamlined M&M).
